# Editorial: Frontiers in oral health: Highlights in oral cancers 2021/2

**DOI:** 10.3389/froh.2022.1083035

**Published:** 2022-12-08

**Authors:** Omar Kujan, Ricardo D. Coletta

**Affiliations:** ^1^ UWA Dental School, The University of Western Australia, Nedlands, WA, Australia; ^2^ Department of Oral Diagnosis and Graduate Program in Oral Biology, School of Dentistry of Piracicaba, Campinas University, Piracicaba-São Paulo, Brazil

**Keywords:** oral cancer, head and neck cancer, special collection, recent advances, editorial

**Editorial on the Research Topic** Frontiers in oral health: Highlights in oral cancers 2021/2

*Oral cancer* is a multi-step tumour associated with diverse presentations and unpredictable outcomes. Regardless of advances in technology and research, the prognosis of oral cancer has not improved over the last decades, which makes this condition a significant health burden that warrants urgent attention in a multidisciplinary manner (1).

This Frontiers Research Topic, *Highlights in Oral Cancers 2021/2*, aimed to shed light on the latest evidence-based findings to answer historically debatable questions in this field.

This Research Topic starts with a review by Philips et al. titled *Preoperative immunotherapy in the multidisciplinary management of oral cavity cancer* to provide insights into current knowledge in the field of oral squamous cell carcinoma (OSCC) immunotherapy. In this review, several hot topics were comprehensively discussed, like immune response, the rationale for preoperative immunotherapy, limitations of immunotherapy, and prospects. A table summarising the results of all relevant clinical trials in the study area was presented to show the latest advances. In conclusion, the authors proposed generating personalised, tumour-specific algorithms based on multiple factors like pathology, response to preoperative immunotherapy, and immune profile to assure optimum oncologic and functional outcomes.

To highlight the inflammatory response's critical role in explaining the defective anti-tumour immunological responses in some OSCC patients, Laliberté et al. conducted a prospective, case-controlled pilot study to characterise OSCC-associated inflammation titled *Characterisation of oral squamous cell carcinoma associated inflammation: a pilot study*. In this study, inflammatory changes in the saliva of 37 OSCC patients were compared with changes in 32 healthy controls with and without periodontitis. Multi-channel flow cytometry with a panel of 12 antibodies was employed to determine the inflammatory cell profile in saliva. Moreover, 30 cytokines/chemokines were evaluated in the saliva samples. Fluorescent immunohistochemistry was undertaken to validate results using a cohort of cancer specimens. This paper showed an increase in OSCC-associated inflammation characterised by specific cytokines (IL-6, IL-8, TNFa, and GMCSF). These findings can be used to design clinical tests that may aid in treatment decision-making, like identifying patients at higher risk of developing disease adverse events and recurrence.

To report the incidence of malignant lymphoid neoplasms in the oral and maxillofacial region, Flores-Hidalgo et al. retrospectively reviewed records of patients diagnosed with lymphoid neoplasms over ten years in two different institutions. This paper is titled *Malignant lymphoproliferative disorders of the oral and maxillofacial region: report of two institutions*. Accordingly, a total of 318 records were retrieved and reassessed according to an updated classification version, of which 138 met the inclusion criteria. Most hematolymphoid malignancies are associated with intra-bony locations. Thus, the authors highlighted the significance of microscopic examination of all tissue samples, even if they present features of persistent periapical lesions after failed root canal therapy.

Further, a paper titled *Preoperative prediction of the aggressiveness of oral tongue squamous cell carcinoma with quantitative parameters from dual-energy computed tomography* was conducted by Yang et al. to propose a preoperative non-surgical tool that helps clinicians and pathologists while diagnosing tongue OSCC cases. Ninety-three out of 161 were included in this study based on clearly specified inclusion criteria and underwent preoperative dual-energy computed tomography (DECT). Specific DECT quantitative parameters were compared to certain pathological characteristics of tongue OSCC. Accordingly, the inter- and intra-observer agreements in evaluating DECT parameters were excellent. Among several DECT parameters, the slope of the spectral Hounsfield unit curve in the venous phase was associated with the highest accuracy (75.3%) in predicting the overall pathological stage. Likewise, the accuracy of the normalised iodine concentration in the venous phase in predicting the histologic differentiation was 75.3%. In conclusion, DECT shows the potential to be a valuable tool for the preoperative evaluation of tongue OSCC aggressiveness.

To answer the controversial question about the concept of resection margins in OSCC surgery, whether adequate or inadequate, Pei et al. conducted a molecular study titled *Risk factors of microscopically tumor-free surgical margins for recurrence and survival of oral squamous cell carcinoma patients*. A total of 235 OSCC patients who underwent surgery over ten years were retrospectively reviewed and included in this study. Two epithelial-to-mesenchymal transition-associated genes (Axin2 and Snail) were immunohistochemically assessed using tissue specimens of surgical margins. The results were validated by developing an *in vitro* model of a knocked-down Axin2 cell line and injecting this cell line into mice. The significant outcomes of this study found that molecular analysis provides a more accurate and objective assessment of surgical margins. In opposition to the histopathological assessment of surgical margins, Axin2 and Snail expressions had independent impacts on the overall survival and recurrence-free survival rates of OSCC patients.

Next, to predict the recurrence of early-stage, HPV-negative head and neck squamous cell carcinoma (HNSCC), a study by Mayhew et al. to identify a gene expression classifier for this purpose was conducted. This study, *Mesenchymal gene expression subtyping analysis for early-stage human papillomavirus-negative head and neck squamous cell carcinoma reveals prognostic and predictive applications*. For training, the authors performed a retrospective genomic analysis of open-source databases, including 418 patients. The classifier was then validated using a different validation cohort. This study demonstrated that mesenchymal subtypes are associated with poor survival rates even in early-stage, lymph node-negative oral cancers. This finding was contrary to the favourable outcomes of the mesenchymal subtype in non-oral cancer cases. The study highlights the significance of gene expression for the treatment decision-making of HNSCC.

Interestingly, several studies assessed the prognosis of patients with oral carcinoma using a mixed population of young and old patients, which were associated with wide discrepancies and heterogeneities, mainly because old patients usually suffered poor systemic conditions. To reduce potential bias, Baba et al. conducted a study that classified patients into three groups instead of two. This study is titled *Comparison between three age-stratified cohorts reveals poor prognosis of young patients with tongue carcinoma*. Two hundred fifty-seven eligible OSCC patients were classified into three groups: young, middle-aged, and older. Accordingly, multivariate analyses found that compared to young patients, there were no differences in overall survival and disease-free survival rates for middle-aged or older patients. However, middle patients were associated with a significant low local recurrence rate. The authors emphasised the importance of categorising patients into various groups according to their age, specifically for patients older than 70 years, while this measure should also be applied to future genomic research.

The seven studies contained in this Research Topic described many relevant aspects for development, progression and treatment of oral cancers, especially highlighting possible therapeutic developments and biomarkers to fight these very aggressive cancers. Such studies are essential towards the development of more effective and predictable therapeutic strategies.
